# Supplementary figures and images for: The luminal ring protein C2CD3 acts as a radial in-to-out organizer of the distal centriole and appendages
Source: PLoS Biol. 2025 Dec 9;23(12):e3003519. doi: 10.1371/journal.pbio.3003519 (PMC12707624; doi:10.1371/journal.pbio.3003519)

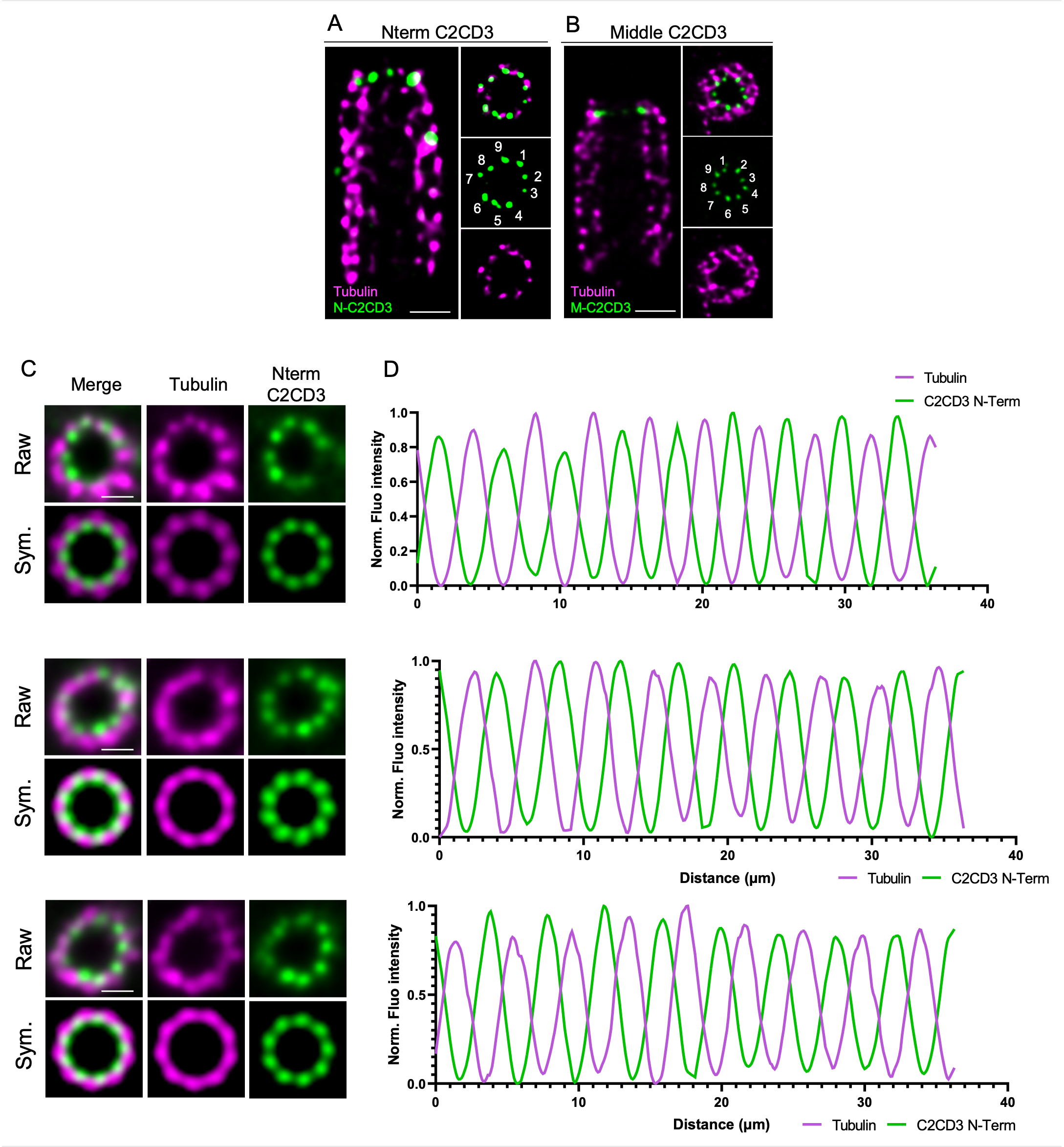

Supplement: S1 Fig — (A, B) Expanded U2OS centrioles using iU-ExM and stained for tubulin in magenta. Nterm C2CD3 (A) or Middle C2CD3 (B) in green. Scale bar: 100 nm corrected by the expansion factor. (C) Additional examples of symmetrization of top-view images with the localization Nter C2CD3 in iU-ExM. Scale bar: 100 nm corrected by the expansion factor. (D) Corresponding intensity plot profiles highlighting the position of C2CD3 N-ter in between microtubule triplets. The detailed statistics of all the graphs shown in the figure are included in the S1 Data file. (TIFF) [file pbio.3003519.s001.tiff]

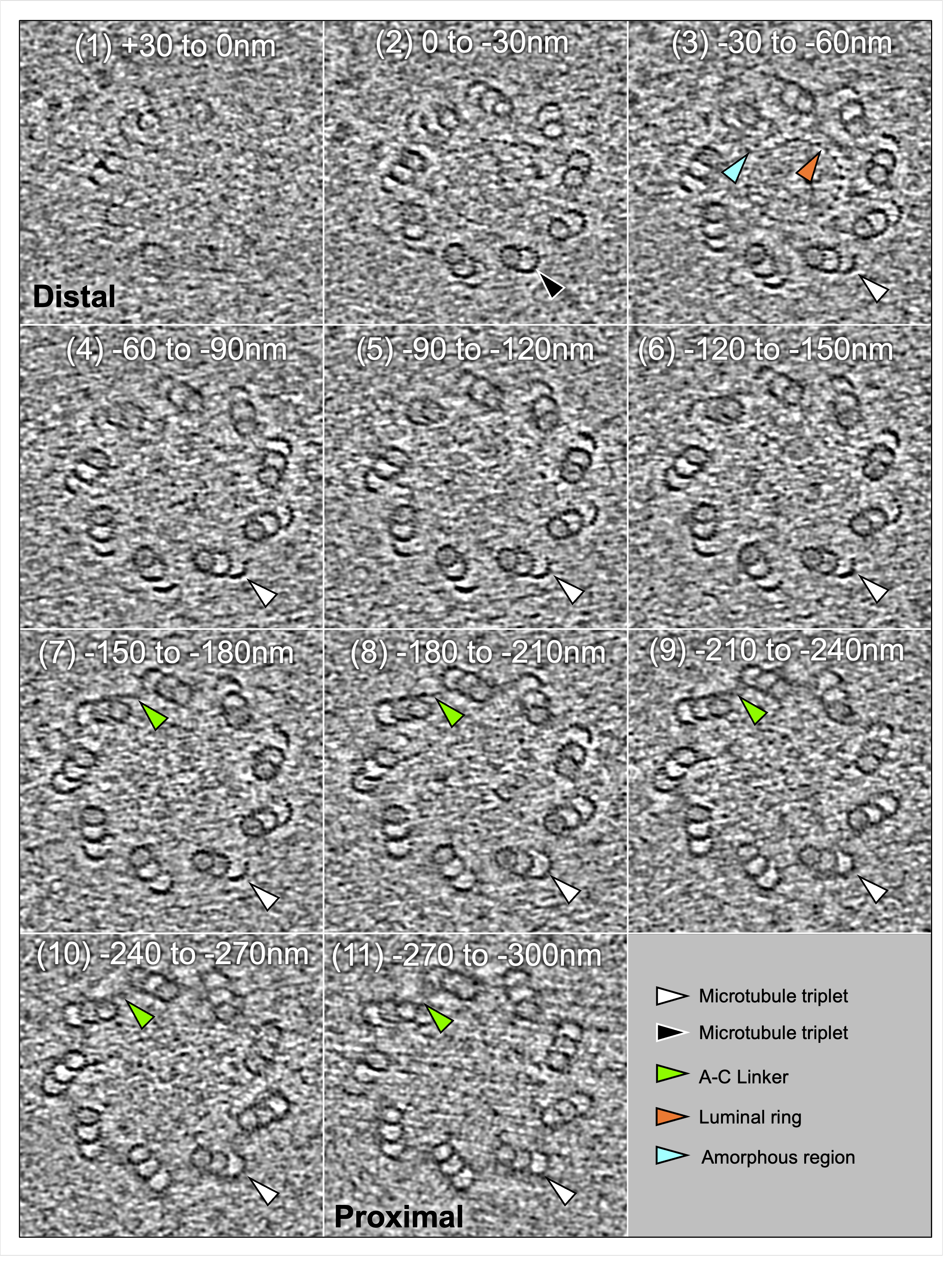

Supplement: S2 Fig — Cryo-tomogram sections of an entire centriole from photoreceptor cells obtained by in situ cryo-electron tomography, shown from the distal (section 1) to proximal end (section 11). Each section represents an average of 31 slices (~30 nm total). A prominent ring-like structure is visible 30–60 nm from the distal tip. (TIFF) [file pbio.3003519.s002.tiff]

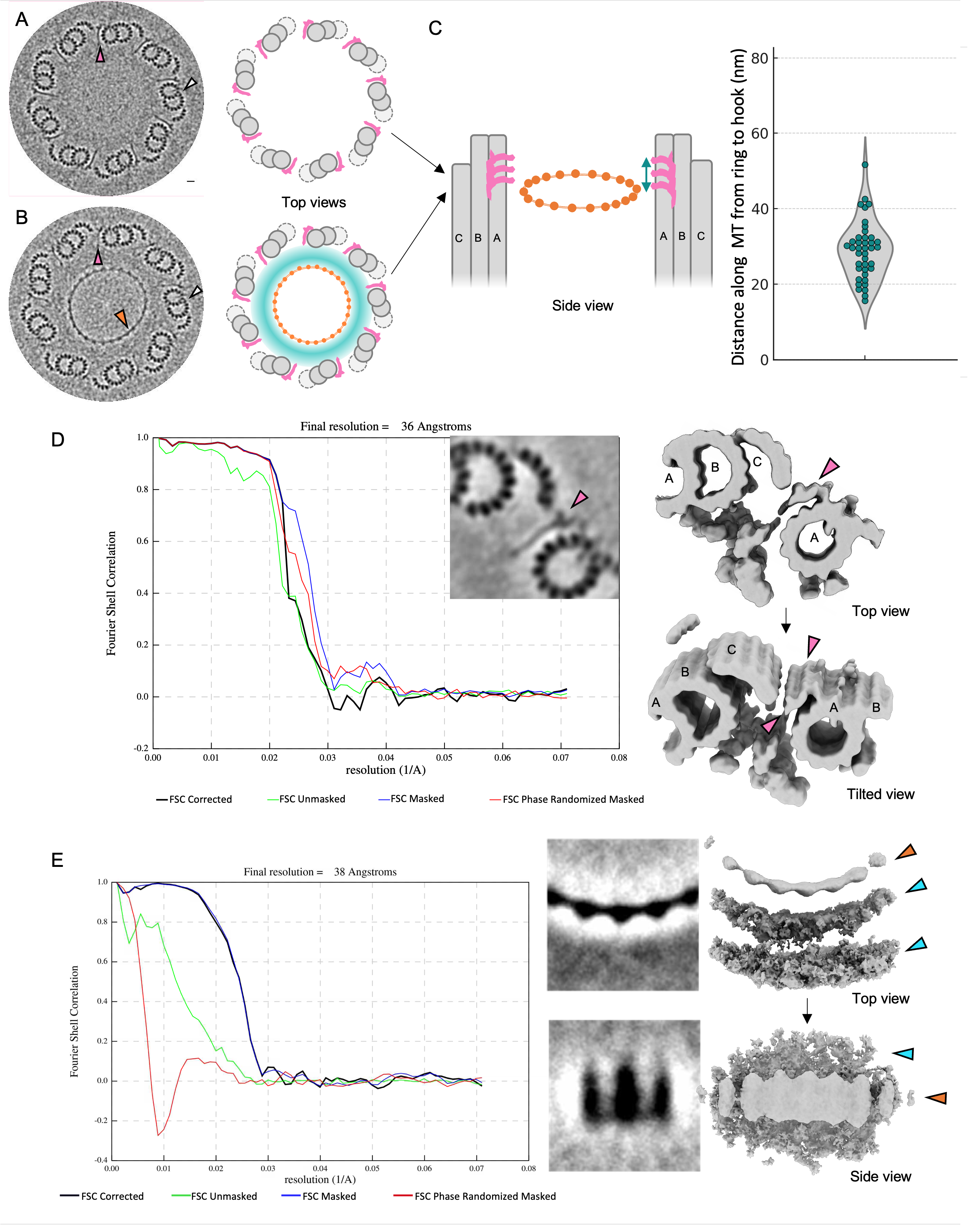

Supplement: S3 Fig — (A, B) Nine-fold symmetrized cryo-tomogram slices of a centriole from mouse tracheal epithelial cells (MTECs). (A) The luminal ring structure is not visible, while the microtubule-associated “hook” structures are observed (pink arrowheads). (B) Both the luminal ring (orange arrowhead) and the hook structures (pink arrowheads) are visible and positioned within the same z-plane, indicating their coplanarity. White arrowheads mark microtubule triplets or doublets [67]. (C) Relative position of the hook structures with respect to the luminal ring. The graph shows the measured distances along the microtubule from the ring structure to the distal end of the hook structures, with an average distance of 28.96 nm ± 7.58 nm. (D) Fourier Shell Correlation (FSC) resolution estimation of the subtomograms average centered on the hook density between microtubule doublets/triplets. Resolution: 36 Å at an FSC cutoff of 0.143. The 3D reconstruction (right) shows continuous hook density along the A-microtubule wall (pink arrowheads). (E) FSC resolution estimation of the subtomograms average centered on the ring structure, containing three node densities. Resolution: 38 Å at an FSC cutoff of 0.143. The corresponding 3D map (right) highlights the amorphous density (blue arrowheads) and the structured ring elements (orange arrowheads). The detailed statistics of all the graphs shown in the figure are included in the S1 Data file. (TIFF) [file pbio.3003519.s003.tiff]

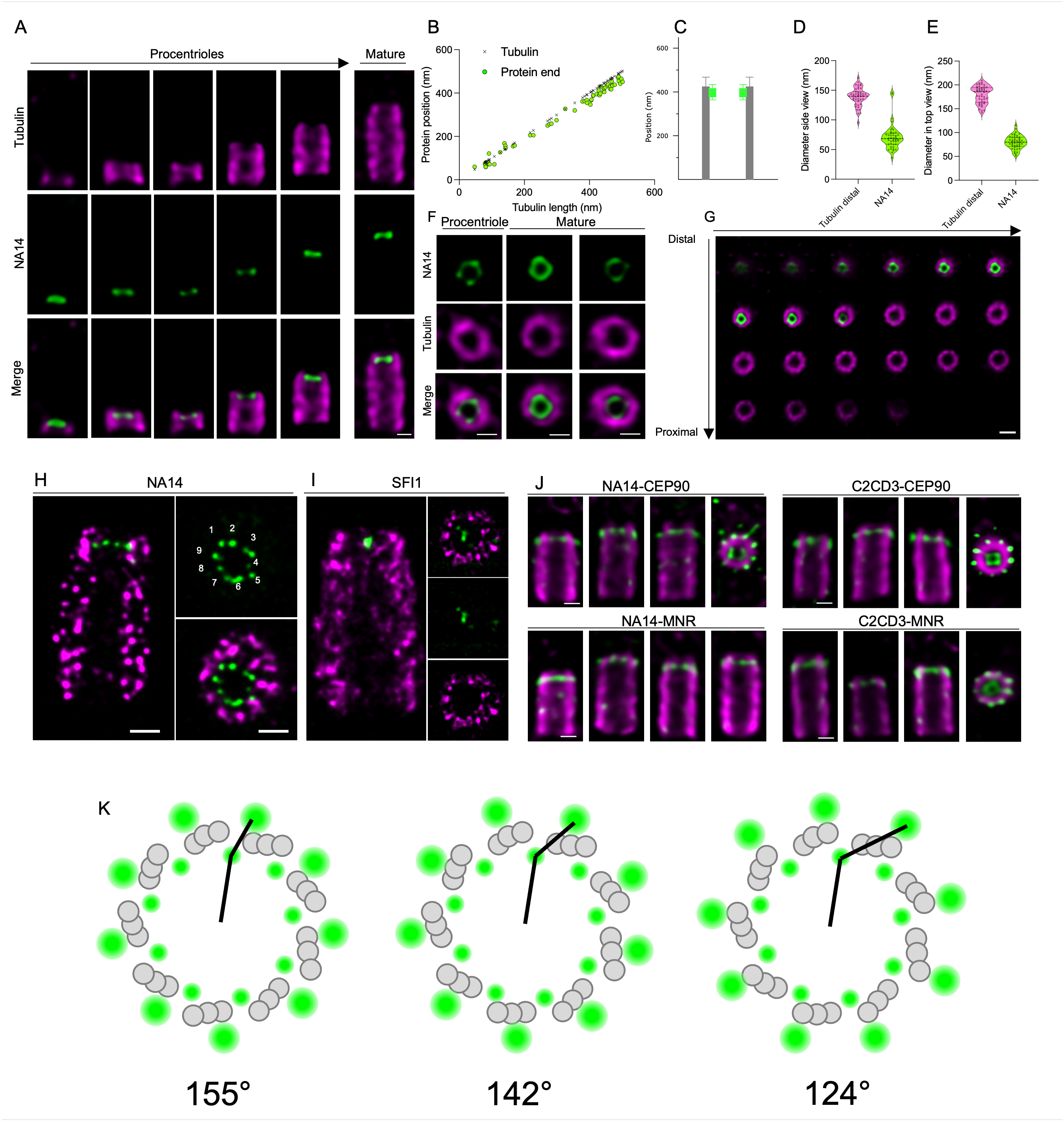

Supplement: S4 Fig — (A) Expanded centrioles from U2OS stained for α/β-tubulin (magenta) and NA14 (green) showing the localization of NA14 during centriole assembly. Scale bars: 100 nm. (B) Quantification of NA14 positioning relative to the distal end during assembly and in mature centrioles. (C) Average position of NA14 in mature centriole according to the tubulin proximal part of the mature centriole. Tubulin length: 438 ± 42; NA14 position: 408 ± 40 nm. (D, E) Distal end diameter measurements from side (D) and top (E) views. Tubulin: 139.21 ± 15.52 nm (n = 43, side), 179.57 ± 18.23 nm (n = 15, top); NA14: 70.85 ± 21.26 nm (n = 43, side), 80.03 ± 13.93 nm (n = 15, top). Data from three independent experiments. (F) Expanded centrioles from U2OS stained for α/β-tubulin (magenta) and NA14 (green) showing the localization of NA14 in top views in procentriole and in mature centrioles. Scale bars: 100 nm. (G) Consecutive z-stack slices of expanded U2OS centrioles stained for α/β-tubulin (magenta) and NA14 (green), highlighting the distal localization of NA14 in top views. Scale bars: 200 nm. (H, I) Expanded U2OS centrioles using iU-ExM, stained for tubulin (magenta) and either in green NA14 (H) or SFI1 (I). Scale bar: 50 nm, corrected for expansion factor. (J) Dual localizations of Mid-C2CD3 with CEP90 and MNR, and NA14 with MNR and CEP90. Proteins of interest in green; tubulin in magenta. (K) Models of dual localization of Mid-C2CD3 with either CEP90 on the A-Microtubule (left), B-microtubule (middle) or C-microtubule (right). For each model, the angle formed by the CEP90 signal relative to the centriole center and the C2CD3 axis is measured and indicated in the figure. The detailed statistics of all the graphs shown in the figure are included in the S1 Data file. (TIFF) [file pbio.3003519.s004.tiff]

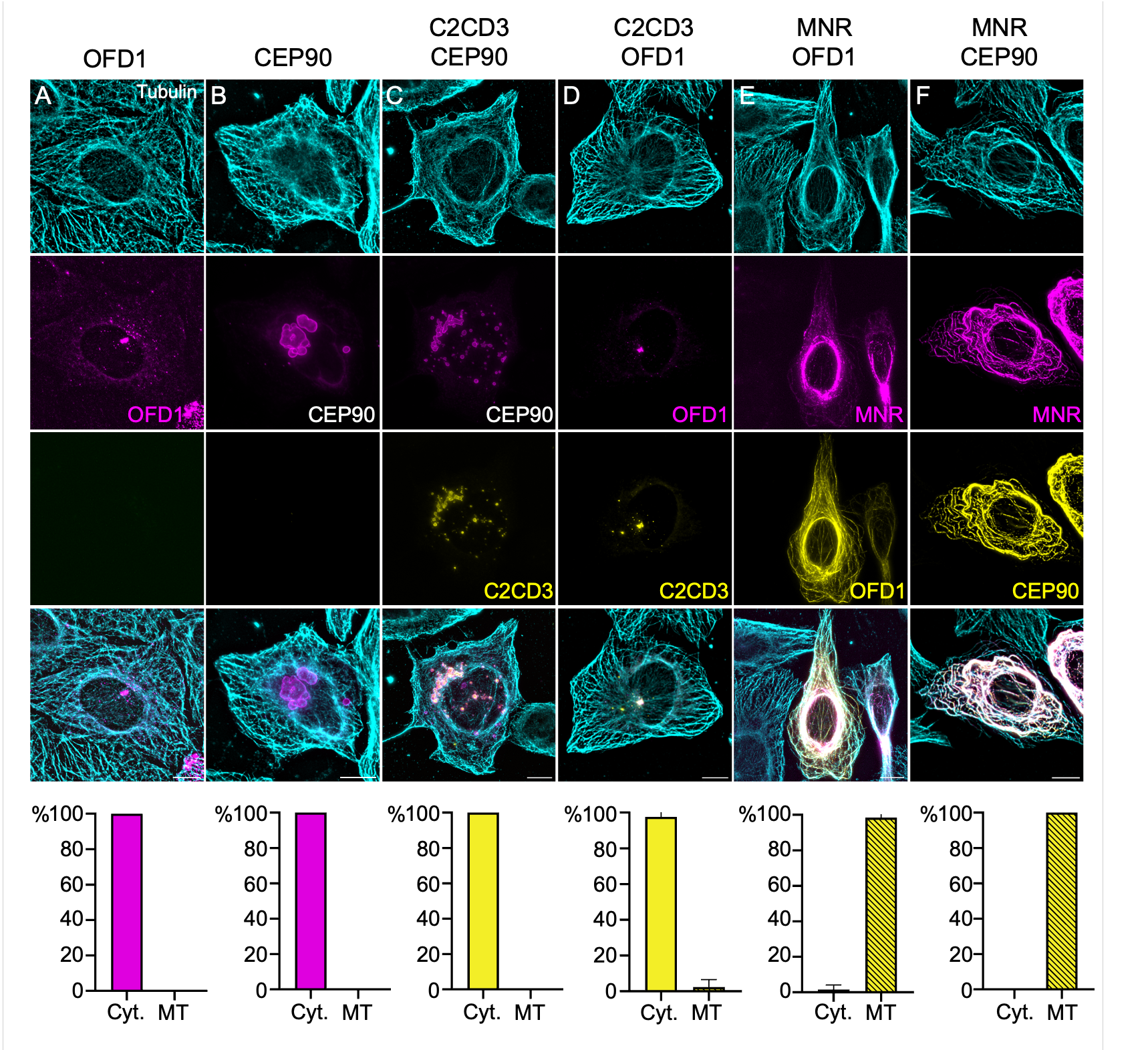

Supplement: S5 Fig — U2OS cells are transfected with (A) mCherry-OFD1, (B) CEP90, (C) C2CD3-GFP and CEP90, (D) C2CD3-GFP, and mCherry-OFD1, (E) MNR-mCherry and mCherry-OFD1, (F) MNR-mCherry and CEP90. When co-transfected with either CEP90 or OFD1, C2CD3 colocalizes in cytoplasmic granules. Both CEP90 and OFD1 are relocalized on microtubules when co-expressed with MNR. Percentage of GFP/mCherry positive cells with proteins localized to cytosol (Cyt.) or microtubules (MT) for each condition. (A) Cyt.: 100; MT: 0, (B) Cyt.: 0; MT: 100, (C) Cyt.: 100; MT: 0, (D) Cyt.: 97.62 ± 4.124; MT: 2.381 ± 4.124, (E) Cyt.: 1.515 ± 2.624; MT: 98.48 ± 2.624, (F) Cyt.: 0; MT: 100. Scale bar: 10 µm Three independent experiments performed for each condition. The detailed statistics of all the graphs shown in the figure are included in the S1 Data file. (TIFF) [file pbio.3003519.s005.tiff]

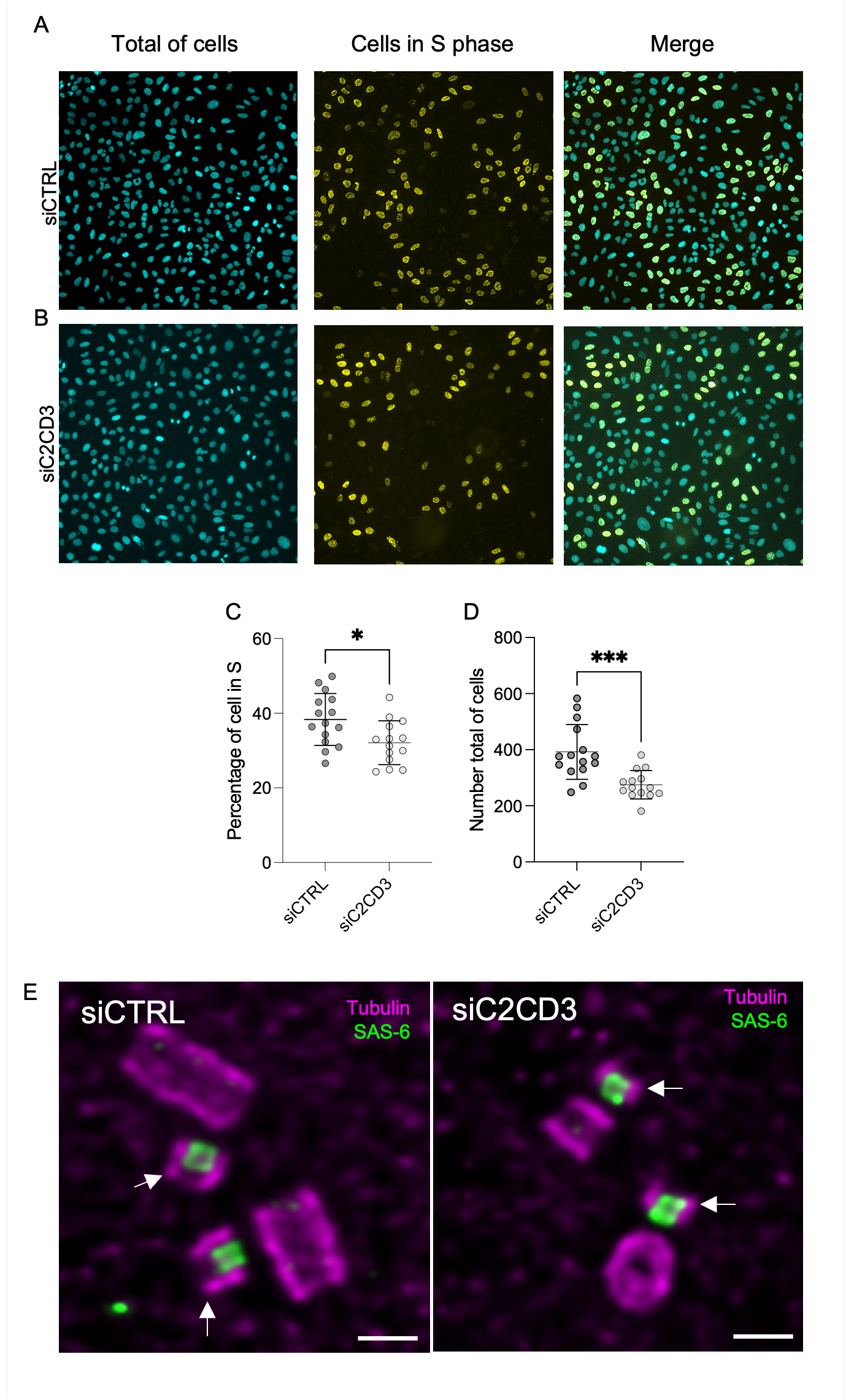

Supplement: S6 Fig — (A, B) Representative fields of U2OS cells treated with siCTRL (A) or siC2CD3 (B), stained with DAPI (cyan) to label nuclei and EdU (yellow) to mark S-phase cells. (C) Percentage of cells in S phase as determined by Click-iT EdU labeling in siCTRL (dark gray) or siC2CD3 (light gray) conditions. siCTRL: 38.32% ± 6.97; siC2CD3: 32.08% ± 5.88. Statistical significance was determined using an unpaired t test (*P = 0.0150). (D) Total number of cells counted per condition (ClickEdU). siCTRL: 392.1 ± 97.49; siC2CD3: 275 ± 50.50. Statistical significance was determined using an unpaired t test (***P = 0.0004). (E) Representative images of expanded centrosomes in siCTRL and siC2CD3-treated cells, stained for tubulin (magenta) and SAS-6 (green). White arrows indicate the microtubule walls of procentrioles, which appear shorter in siC2CD3-treated cells compared to controls, despite having similar cartwheel (SAS-6) lengths. The detailed statistics of all the graphs shown in the figure are included in the S1 Data file. (TIFF) [file pbio.3003519.s006.tiff]
