# Supplementary material for: The luminal ring protein C2CD3 acts as a radial in-to-out organizer of the distal centriole and appendages
Source: PLoS Biol. 2025 Dec 9;23(12):e3003519. doi: 10.1371/journal.pbio.3003519 (PMC12707624; doi:10.1371/journal.pbio.3003519)
Supplement: Attachment — Submitted filename: rebuttal-Bertiaux_el_al.docx [file pbio.3003519.s010.docx]

PLOS Biology Submission (PBIOLOGY-D-25-01923R1) - [EMID:6c9bbee18df299bc]

We would like to thank the editor and the Reviewers for their comments and suggestions to further improve the manuscript. We have made all efforts to address the comments and suggestions and detail our replies in the point-by-point response below. Highlight changes in the text are shown in blue for the sake of clarity.

REVIEWS:

**Reviewer #1**: Centrioles are nine-fold microtubule-based structures playing an important role in cell division, cellular polarity and as basal bodies, represent sort of ciliary anchors. Appendages important for centriole docking to the plasma membrane and ciliogenesis are present at the distal ends.   As stated by the authors, C2CD2 has been suggested to play a role for recruitment of protein complexes playing an important role for appendage assembly and dysfunction of C2CD3 results in a ciliopathy phenotype.  It has also been previously shown that C2CD3 appears as a ring-like structure forming the luminal distal ring (LDR) complex together with other proteins and that C2CD3 proteins are arranged in a 9-fold symmetrical arrangement positioned near the A-tubule. This suggested a scaffolding role for C2CD3 however the precise architectural mechanism by which C2CD3 establishes the nine-fold symmetry of distal appendage assembly has remained unclear. 

In this paper, the authors investigate the precise molecular organisation of the distal centriolar region using advanced imaging techniques including U-ExM and cryo-electron tomography. They confirm the previously proposed nine-fold  symmetry and suggest that the C2CD3 C-terminus localizes to the lumen while the C2CD3 N-terminus locates between microtubule blades with a"hook" connector between microtubule blades near the C2CD3 N-terminus. This explains previously reported discrepancy of diameters of 80-90 nm found using U-ExM, and a 146.4 nm ring with nine distinct puncta using U-ExM  combined with STORM as the smaller diameter was seen using an antibody detecting the C-terminus of C2CD3 whereas the larger diameter was seen using an antibody detecting the middle part of C2CD3.  The authors map out precise positions of C2CD3 and other proteins part of the appendage protein complexes, C2CD3 might bridge the centriole lumen to the external DISCO complex, forming an in-to-out
connection through the space between microtubule triplets/doublets.

Using displacement assays using overexpression, they show that C2CD3 does not bind microtubules when overexpressed alone but when co-expressed with MNR and that overexpression of MNR with C2CD3, C2CD3/CEP90, and C2CD3/OFD1/CEP90 led to increased microtubule localization, suggesting a cooperativity in the binding. They further show C2CD3 interacts with appendage-anchoring proteins MNR and CEP90, and contributes to a broader distal complex including SFI1, Centrin-2, CEP135, and NA14/ SSNA1 with NA14/ SSNA1 co-localizing with the C2CD3 C-terminus. 
They also found that depletion of C2CD3 using siRNA results in shorter centrioles with abnormal CEP97 and CEP162 localization while core structural features of the proximal and middle centriole regions are intact. From this they conclude C2CD3 plays an important role for distal centriole architecture as well as microtubule cap composition.
Overall, this is a very well written manuscript with superb imaging data and 3D reconstructions, providing novel insights into the structural organisation of centrioles and the precise role of C2CD3. I feel this will be interesting to a broad readership and only have very few comments.

We thank the reviewer for the very positive evaluation of our manuscript.

1.      siRNA knockdown: I couldn't find data on protein expression in control vs siRNA cells. This should be added to the supplement.

We thank the reviewer for this suggestion. We attempted to detect endogenous C2CD3 by Western blot using three commercially available antibodies and under several blotting conditions (image below). However, the expected band at ~250 kDa was not visible, preventing reliable quantification of C2CD3 levels in control or siRNA-treated cells. Nevertheless, the specific loss of the fluorescent signal corresponding to C2CD3 at centrioles following siRNA treatment, consistent with previously published data, confirms the efficiency and specificity of the knockdown. We have added a note in the Materials and Methods section indicating that C2CD3 protein levels could not be assessed by Western blot due to the lack of detectable signal under control conditions.

2.      Figure S6 C suggest a statistically significant reduction of cells in S Phase (something like 38% vs 32%) in C2CD3 siRNA cells vs siRNA controls while in figure S6 E and F, bars suggest that in both groups, less than 20% were in S Phase with no striking difference. Does this difference between C and E/F stem from different methods used and is there a statistically significant difference between the two groups in E and F, also with regards to other cell cycle phases?

We thank the reviewer for this valuable comment.

Indeed, these panels represent two distinct approaches: Figure S6C was generated using Click-EDU incorporation followed by manual counting, whereas Figures S6E–F were obtained through automated microscopy-based analysis from our facility. The apparent discrepancy arises from differences in sensitivity and quantification between the two methods. Statistical analysis of Figures S6E–F revealed no significant differences between control cells and C2CD3 siRNA–treated cells across all phases of the cell cycle.

However, we recognize that the automated method underestimates the proportion of cells in S phase and overestimates those in G2 phase, which should typically represent about 10–20% of the population. Although both samples were processed under identical conditions and the results are therefore comparable, we decided to remove this analysis, as it does not provide additional information beyond the Click-EDU data and could lead to confusion. The text has been corrected accordingly.

Reviewer #2 (Maxence Nachury, identifies himself): This manuscript presents an unparalleled combination of iterative expansion microscopy and cryo-electron tomography to paint the spatial organization of C2CD3 from inside the centriole to the inter-MT space of the centriolar barrel.

The manuscript explores the  distal luminal ring complex, a structure first visualized in the 1970s, but with no molecular characterization to speak of. By carefully matching the locations of C2CD3-Cterm determined by U-ExM and those of the distal luminal ring complex mapped by cryoET, the authors propose that C2CD3 forms part of the distal luminal ring complex.

A major advance of the manuscript is the modeling of the distal ring structure from cryoET data, which consists of a 100nm ring with 27 nodes connected to nine-fold symmetrical amorphous linkers and hook anchored onto the A microtubule.

The rigor, precision and breadth of the work allows the authors to assemble a persuasive model for C2CD3 spanning the distal luminal ring to the DISCO complex on the outside of the centriole by fitting through the microtubule triplet blades.

Adding to the novelty, the authors identify the protein NA14 as part of the distal luminal ring.

The graphical summaries are very helpful to orient the non-specialist through the protein landscape of the distal centriole.

I have no major issue with the manuscript and strongly recommend publication.

Minor: scale bars are missing from several panels (1L, 3E, leftmost panel in 3A, bottom panels in 3D,

4D/E/F/H/I/J/L/M/N/P/Q, 5G-L, S1A-C, S4A, S4F)

We thank the reviewer for noticing this oversight. We have now added all the missing scale bars.

I was confused about the graph in 3J. Shouldn't the bar be colored yellow if they depict C2CD3

distribution?

We agree with the reviewer’s comment and have corrected the figure as suggested.
